# Psychosocial correlates of flourishing in the late lifespan

**DOI:** 10.1007/s40520-024-02703-z

**Published:** 2024-03-14

**Authors:** Maria Chiara Fastame, Cristina Manca, Ilaria Mulas, Marilena Ruiu

**Affiliations:** Department of Pedagogy, Psychology, Philosophy, University of Cagliari, Via Is Mirrionis 1, 09123 Cagliari, Italy

**Keywords:** Flourishing, Aging, Psychological hardiness, Cognitive failure, Satisfaction, Resilience, Eudaimonic well-being

## Abstract

**Background:**

Flourishing is a primary dimension of psychological well-being that contributes massively to the development of an active, purposeful, and respectful life, full of meaning, values, and personal interests that nurture social ties.

**Aims:**

This study primarily intended to examine the contribution of satisfaction with family relations, resilience, metacognitive efficiency, and crystallized intelligence in predicting a flourishing measure in cognitively healthy older adults. Moreover, the impact of gender was investigated on flourishing, satisfaction with family ties, resilience, and metacognitive efficiency.

**Methods:**

One hundred and eighty 65–94-year-old community dwellers were recruited in Sardinia (Italy). Participants self-rated their flourishing, satisfaction with their family connections, psychological hardness (i.e., a dimension of resilience), and cognitive function, whereas global cognitive efficiency and vocabulary were assessed through two internationally validated objective tests.

**Results:**

A hierarchical regression analysis revealed that 30% of the variance in the flourishing condition was explained by satisfaction with family ties, resilience, and metacognitive efficiency. In addition, males exhibited higher flourishing and satisfaction with family ties than females, and the former group also reported being more autonomous and acting proactively to influence its destiny.

**Conclusion:**

Emotional support and rewarding relations with family members, the ability to face stressful events, and a good perception of one’s cognitive efficiency play a crucial role in promoting flourishing in late adulthood.

## Introduction

The aging of the world’s population is a well-known social phenomenon, such that by 2050 people over 65 years are expected to be 1.6 billion, that is, 16% of the global population [1]. From an applied perspective, this is a meaningful concern since advancing age is usually associated with the need for greater functional and psychological assistance. That is, the aging population represents a challenge to be faced by the healthcare systems to guarantee adequate levels of quality of life but at the same time to contain the costs allocated for the health of older individuals (e.g., [2]). To pursue these goals, it is crucial to elucidate the factors contributing to successful aging. Despite the lack of a consensual definition, assuming a biopsychosocial viewpoint, successful (also called optimal, positive, healthy, or productive) aging refers to a multidimensional construct reflecting the combination of cognitive and functional resources enabling one’s independence, engagement in cognitively and socially stimulating leisure activities, social participation, presence of significant social ties, neuroplasticity compensating the effects of age-related decline, and a wide range of positive psychosocial features (e.g., wisdom, personal growth, satisfaction with life, optimism) being adopted to contrast the impact of age-related diseases and life stressors [3], see also [4].

A robust tradition of research pinpoints the strong association between successful aging and psychological well-being (e.g., [5, 6]). The latter is a multifaceted resource encompassing the hedonic component (i.e., which is strictly related to happiness and the promotion of pleasure, satisfaction, and enjoyment) and the eudaimonic one (which is aimed at developing one’s potentialities and values, promoting personal growth, autonomy, self-realization) [7]. It is well-established that one crucial facet of psychological well-being is flourishing, of which different definitions have been provided. For instance, it has been postulated that flourishing implies a valuable and meaningful life characterized by positive and supportive relations, self-acceptance, social participation, self-confidence, and optimism [8]. Consistent with this view, Hone et al. [9] argued that flourishers are vital and focused on the pursuit of expressing their human potential under their more genuine and true self. Therefore, older people exhibiting higher levels of flourishing embrace life [10] and interact with others (e.g., they support others and are supported) to develop a sense of achievement and purpose [11]. Besides, as highlighted by Keys [12], flourishing is a condition of mental health that does not merely imply the absence of disabilities or illnesses but rather the construct encompasses emotional well-being (e.g., happiness, interest in life), psychological well-being (i.e., self-acceptance, personal growth, purpose in life, autonomy, positive relations with others, environmental mastery), and positive social functioning (e.g., social participation and fostering enduring relations), which, in turn, enhance the quality of life.

Various findings corroborate the idea that more developed flourishing in late lifespan is associated with better physical and mental health-related outcomes, and with a more socially oriented life [13]. Moreover, a longitudinal study conducted using the data of the Terman Life Cycle Study revealed that sociability in childhood predicted flourishing (i.e., a multidimensional construct defined primarily by family ties, community relationships and then by life satisfaction, positive affect, and achievement) in midlife (average age 40), which, in turn, was prospectively associated with lower mortality risk [14].

There is also evidence that older males exhibit higher flourishing than females, perhaps because the latter group usually also reports higher anxiety and depression signs [12, 15]. Moreover, it has also been found that age, financial condition, marital status, and living alone do not predict flourishing in the late adult lifespan [15].

A further stream of research highlighted the relevance of social capital to enhancing psychological well-being in the late lifespan (e.g., [11, 16, 17]). For instance, research conducted in 16 countries revealed that primarily family-based relationships (e.g., with the partner, children, and finally other relatives) and then non-family ties predicted a measure of eudaimonic and hedonic well-being in a sample of approximately 30.000 65–104-year-old participants [17]. These outcomes are reinforced by a further study, according to which older individuals living alone and with a very poor social network reported lower flourishing assessed through the tool developed by Diener et al. [18], whereas those who had a good social network, regardless of living alone or with others, exhibited better flourishing [19]. In addition, a recent study conducted by Kohn et al. [20] supports the ‘paradox of aging’ [21], that is, despite physical and cognitive decline being more evident in the last 2 decades of life, octogenarians and older individuals exhibited preserved flourishing which was enhanced by happiness, personal mastery, and prosocial behaviors focused on providing help and support to others. Extending this, studies conducted in areas of exceptional longevity (i.e., the so-called ‘Blue Zone’) documented that older people who stated to be satisfied with their family ties reported fewer depressive signs and loneliness, and higher perceived psychological well-being than people having less social functioning (e.g., [22–24]). Consistent with this, a recent investigation conducted with older people living in the Sardinian Blue Zone revealed significant and positive associations between a well-known measure of flourishing developed by Diener et al. [18] and two indexes of satisfaction with family and non-family ties [25]. However, the authors did not find any significant role of satisfaction with both types of social connections in predicting the flourishing measure.

Moreover, Fastame and Melis [26] documented that the self-reported measure of Flourishing developed by Diener et al. [18] was negatively associated with the efficiency of crystallized intelligence and with a measure of subjective cognitive function (i.e., the Cognitive Failure Questionnaire by [27], see the Materials section) taken in a sample of 65–94-year-old community dwellers. Partially consistent with these outcomes, Kohn et al. [20] found a significant association between flourishing and self-reported cognitive complaints (i.e., assessed through the Cognitive Failure Questionnaire) only in a sample of 60–79-year-old individuals but not in an older group (i.e., ≥ 80 years of age). Overall, the aforementioned findings suggest that less cognitively healthy older people and those complaining more about the efficiency of their mind tend to disinvest in terms of living a life full of purposes and values (i.e., languishing in life), as well as tend to be less vital.

Finally, further studies suggest that there is an interplay among successful aging, psychological well-being, and resilience in later life [21]. Specifically, resilience is a personality trait that allows older people aging well to face adversities (e.g., the occurrence of illnesses and bereavement) and to recover from such stressful events [28]. Consistent with this, the definition proposed by Ryff and Singer [10] of resilience as “the maintenance, recovery, or improvement in mental or physical health following challenge” (p. 20), points out that the construct reflects a resource to engage successfully with adverse life experiences and difficult events characterizing the late adulthood and to preserve or even foster one’s flourishing. A recent meta-analytic review documented that resilience conceived as a personality trait was not correlated with age in late adulthood, as well as no differences in that indicator of positive adaption to stress were found between old males and females [29]. Moreover, it has been documented that older individuals reporting higher resilience are less depressed and anxious, and self-report better levels of psychological well-being and quality of life [29]. Consistent with this, a study conducted by Fastame et al. [22] with a sample of older people aging successfully and living in the Sardinian Blue Zone revealed that a dimension of resilience, the so-called optimal regulation (i.e., it refers to a set of behavioral and temperamental characteristics engaged to manage negative emotionality related to anger, anxiety, and disengagement) contributed to predicting a measure of psychological well-being encompassing flourishing (i.e., the Warwick–Edinburgh Mental Well-Being Scale).

However, to date, no studies have concurrently explored the role played by satisfaction concerning one’s relations with relatives, resilience assessed in terms of psychological hardiness (see the Material section), metacognition, and cognitive efficiency in shaping flourishing in late adulthood. Moreover, thus far, to our knowledge, no studies have been conducted in Italy to evaluate psychological hardiness in the last 2 decades of life. In our opinion, this issue needs to be disentangled since there is evidence only of their single contribution as promoters of psychological well-being in late adulthood and of successful aging. Therefore, to address this knowledge gap, the current investigation intended to examine: (1) the associations between psychological flourishing, age, self-assessed satisfaction with family relationships, resilience, cognitive complaints, and crystallized intelligence in late adult lifespan; (2) whether the abovementioned psychological measures predicted a measure of flourishing that was self-assessed by older individuals; (3) the impact of gender on the abovementioned dimension of eudaimonic well-being, resilience, and metacognitive efficiency in late adulthood. Thus, consistent with previous studies, the following hypotheses were yielded: (1) significant associations were expected between flourishing and satisfaction with family relationships (e.g., [22, 25]), resilience [22, 29], and vocabulary [26], respectively, (2) flourishing was not expected to be associated with age [15], (3) males were expected to exhibit higher flourishing [12, 15], (4) no gender differences were expected in the self-assessment of cognitive function [30]. In contrast, no specific hypotheses can be formulated on the role of satisfaction with family ties in predicting flourishing in late adulthood, since evidence is controversial [17, 25]. Similarly, due to the lack of univocal evidence, no specific hypotheses are proposed on the association between flourishing and perceived cognitive function in late adulthood [20, 26]. In addition, no specific hypotheses on the impact of gender on resilience are drawn, because according to Fastame et al. [22], males are more resilient than females, but this was not found by Färber and Rosendahl [29]. Finally, due to the lack of previous findings, no further a priori predictions were provided.

## Methods

### Participants

One hundred and eighty 65–94-year-old adults, 77 males and 103 females (M_age_ = 77.4 years, SD = 6.10 years), were recruited in Sardinia (Italy). To participate in the study, respondents had to be community dwellers and cognitively healthy (i.e., a score to the Mini-Mental State Examination, MMSE, ≥ 24). Gender (χ^2^ 3.756, df = 1, *p* = 0.053) and educational attainment (i.e., ≤ 8 years of formal schooling vs. > 8 years of formal schooling) (χ^2^ 3.2, df = 1, *p* = 0.074) were counterbalanced across the participants.

## Materials

The following battery of tools was administered:

The Mini-Mental State Examination (MMSE, [31]) was proposed as a screening test for global cognitive efficiency. Participants exhibiting a score adjusted for age and educational attainment < 24 were excluded for suspected cognitive decline.

The socio-demographic interview by Fastame [32] was used to collect some information on the lifestyle and socio-demographic characteristics of the participants.

The Flourishing Scale by Diener et al. [18] was used to evaluate eudaimonic well-being. This questionnaire encompasses eight items that are designed to rate distinct dimensions of eudaimonic well-being facets, such as relationships with others, optimism toward one’s future, and having a gratifying and purposeful life. Specifically, for each statement, the participants had to self-assess their degree of agreement on a Likert scale ranging from 1 (Complete agreement) to 7 (Complete disagreement, maximum total score = 56). Following Hone et al. [8], a score ≥ 48 reflects a high level of flourishing.

The self-assessed personal satisfaction about family relationships index used elsewhere [16] encompasses three items, in which each respondent was invited to evaluate his/her degree of satisfaction concerning his/her relationships with family members during the previous week along a Likert scale ranging from 0 (i.e., lack of satisfaction) to 10 (i.e., maximum satisfaction).

The 15-item Dispositional Resilience Scale (DRS-15, [33]; Italian validation, [34]) was used as a measure of a personality trait, namely the psychological hardiness, which is a crucial resource of resilience to cope with stressful and aversive events. People reporting greater hardiness exhibit a stronger sense of life, they control accurately what they do, feel involved with their work, and are more responsive to the changes occurring in their lives. For each of the 15 statements, the participants were invited to self-rate the degree of agreement using a Likert scale ranging from 0 (not true at all) to 3 (completely true). The maximum total score is 45. Apart from a total score of resilience (i.e., DRS-15-tot score), the tool provides three distinct indices: control which refers to the ability to influence one’s own life (e.g., ‘if you work hard, you can pursue your goals’), commitment that reflects a sense of meaning and purpose to one’s self, others, and job occupation (e.g., ‘the majority of my life is occupied doing meaningful things’), and challenge that implies the idea that life changes are an opportunity of personal growth (e.g., ‘I think that changes in my daily routine are interesting’). This tool was proposed since it is one of the two most used instruments to evaluate the construct of hardiness and because previous studies conducted with older participants showed that it is a reliable and valid measure (e.g., [35–37]). In this regard, it has been documented that participants exhibiting more psychological hardiness also reported a few depressive symptoms (e.g., [35, 37]).

The Italian version [30] of the Cognitive Failure Questionnaire (CFQ) by Broadbent et al. [27] was used as a measure of metacognitive efficiency (or self-perceived cognitive function) since this tool evaluates the occurrence of cognitive errors. This questionnaire encompasses 25 items referring to very common situations experienced in daily life (e.g., “Do you forget the name of people”). Considering the past 6 months, for each statement, the respondents were invited to self-assess the occurrence of the problem using a Likert scale ranging from 0 (i.e., never) to 4 (i.e., very often, maximum total score = 100). Following De Beni et al. [30], a score ≤ 22.5 indicated high self-perceived metacognitive efficiency.

The Italian version [38] of the Vocabulary Subtest of the Wechsler Adult Intelligence Scale (WAIS, [39]) was designed as an objective measure of crystalized verbal intelligence. Specifically, respondents had to define the meaning of 35 words by recalling this lexical and semantic information from their long-term memory. Based on the degree of clarity, exhaustiveness, and correctness, each response was evaluated following the criteria suggested by Wechsler [39], and assigning a score ranging from 0 to 2 (maximum total score = 70).

### Ethics statement

The study was conducted in accordance with the ethical standards of the institutional research committee and with the 1964 Helsinki Declaration and its later amendments. Written informed consent was given by all participants before participation.

### Procedure

Each participant was individually examined in a quiet room of his/her own house. If the MMSE score was ≥ 24, the preliminary interview was proposed, and then the presentation order of the further tests was counterbalanced across the participants, according to the Latin Square procedure. To avoid the fatigue effect, the examiner read aloud each statement and recorded the answers provided by the participant on the response sheet. Each experimental session lasted approximately 70 min.

### Statistics

All analyses were performed using IBM SPSS Statistics version 24 (SPSS Inc., Chicago, IL, USA). Statistical significance was set to *p* values < 0.05. Descriptive statistics were performed to explore the socio-demographic characteristics of the respondents. Pearson product–moment correlations were calculated to examine the nature of the relationships among eudaimonic well-being, age, satisfaction with family ties, resilience, perceived metacognitive efficiency, crystallized intelligence, and global cognitive function. Based on the outcomes of the correlational analyses, a hierarchical regression analysis was performed to explore whether the abovementioned psychological measures predicted the flourishing index. Finally, a series of *t *tests was conducted to investigate the impact of gender on flourishing, satisfaction with family relationships, resilience, and perceived metacognitive efficiency.

A priori power analysis using the G-power program [40] revealed that to perform the correlational analyses a convenient sample of 112 participants would be necessary, when r = 0.3, power = 0.9, with alpha at 0.05. Besides, to carry out a regression analysis using 6 predictors, with 90% power, alpha at 0.05, and moderate effect size (f^2^ = 0.20), an a priori power analysis established that 138 participants would be needed. Finally, to perform a two-tailed *t *test with independent samples, with 90% power, alpha at 0.05, and a medium effect size d = 0.5, a sample of at least 172 participants would be necessary.

## Results

Table 1 summarizes the results of Pearson’s correlations among eudaimonic well-being, age, satisfaction with family relationships, resilience, self-reported metacognition, vocabulary, and global cognitive function. As can be seen, the examined variables were not highly correlated with each other (r $\ge$ 0.9).

**Table 1:** Person’s correlation matrix among eudaimonic well-being (i.e., Flourishing), age, satisfaction with family ties (i.e., Family-satisf), global score of resilience (i.e., DRS15-tot score), resilience expressed in terms of commitment (i.e., DRS15-commitment), resilience expressed in terms of control (i.e., DRS15-control), resilience expressed in terms of attitude to face the challenges (i.e., DRS15-challenge), self-perceived cognitive failures (i.e., CFQ), crystallized intelligence (i.e., vocabulary), and general cognitive function (i.e., Mini-Mental State Examination, MMSE)

	1. Flourishing	2. Age	3. Family-satisf	4. DRS15-tot score	5. DRS15-commitment	6. DRS15-control	7. DRS15-challenge	8. CFQ	9. Vocabulary	10. MMSE
1	–									
2	0.057	–								
3	0.374***	0.154*	–							
4	0.317***	− 0.026	0.172*	–						
5	0.474***	0.011	0.215**	0.68***	–					
6	0.426***	− 0.013	0.290***	0.727***	0.463***	–				
7	− 0.148*	− 0.038	− 0.084	0.645***	0.089	0.079	–			
8	− 0.445***	− 0.168 *	− 0.179*	− 0.166*	− 0.216**	− 0.292***	0.114	–		
9	− 0.176*	− 0.237 **	− 0.112	− 0.166*	− 0.320***	− 0.110	0.030	0.173*	–	
10	0.07	− 0.094	− 0.003	0.077	0.106	0.050	0.025	− 0.185	− 0.192	–

*p < 0.05, **p < 0.01, ***p < 0.001

Next, based on the results of the correlational analyses, a hierarchical regression analysis performed by ordinary least squares estimation was carried out to examine whether satisfaction with family ties, the global resilience measure, perceived cognitive failures, and the efficiency of crystallized intelligence predicted the eudaimonic well-being index. Specifically, the self-reported satisfaction with family ties measure was entered in Step 1, the total resilience score was entered in Step 2, the self-reported cognitive failures measure was entered in Step 3, and the vocabulary score was entered in Step 4. The measure of flourishing was used as the dependent variable. Preliminary analyses were performed to ensure no violation of the assumptions of normality, linearity, and homoscedasticity. Besides, tests to see if the data met the assumption of collinearity indicated that multicollinearity was not a concern (satisfaction with family ties, tolerance = 0.95, VIF = 1.06; resilience, tolerance = 0.92, VIF = 1.08; cognitive failures, tolerance = 0.94, VIF = 1.07; vocabulary, tolerance = 0.92, VIF = 1.09). Table 2 presents the results.

**Table 2:** Summary of hierarchical regression analysis for variables predicting eudaimonic well-being (i.e., Flourishing)

Dependent Variable	Predictor	*B*	95% CI for B		*SE B*	*β*	_Adjusted R_^2^	ΔR^2^
			LL	UL				
Flourishing	Step 1						0.14	0.14***
	Constant	38.856***	35.467	42.245	1.716			
	Family-satisf	1.012***	0.619	1.405	0.199	0.37		
	Step 2						0.18	0.04**
	Constant	32.440***	27.234	37.645	2.636			
	Family-satisf	0.908***	0.520	1.296	0.197	0.33		
	DRS15-tot score	0.257**	0.096	0.418	0.082	0.23		
	Step 3						0.30	0.12***
	Constant	39.673***	34.232	45.115	2.756			
	Family-satisf	0.750***	0.388	1.111	0.183	0.27		
	DRS15-tot score	0.222**	0.073	0.370	0.075	0.19		
	CFQ	− 0.17**	− 0.227	− 0.107	0.030	− 0.37		
	Step 4						0.305	0.005
	Constant	41.055***	35.112	46.997	3.009			
	Family-satisf	0.739***	0.377	1.101	0.183	0.27		
	DRS15-tot score	0.204**	0.052	0.356	0.077	0.18		
	CFQ	− 0.161***	− 0.222	− 0.101	0.031	− 0.354		
	Vocabulary	− 0.025	− 0.070	0.019	0.022	− 0.08		

Satisfaction with family relationship (i.e., Family-satisf), resilience (i.e., DRS-tot score), self-perceived cognitive failures (i.e., CFQ), and crystallized intelligence (i.e., vocabulary) measures were used as the predictors

CI = confidence interval; LL = lower limit; UL = upper limit

**p < 0.01 ***p < 0.001

Finally, the impact of gender on eudaimonic well-being, satisfaction with family ties, resilience, and perceived cognitive failure measures is illustrated in Table 3.

**Table 3:** *t *tests performed to examine the effect of gender on eudaimonic well-being (i.e., Flourishing), satisfaction with family relationships (i.e., Family-satisf), global score of resilience (i.e., DRS15-tot score), resilience expressed in terms of commitment (i.e., DRS15-commitment), resilience expressed in terms of control (i.e., DRS15-control), resilience expressed in terms of attitude to face the challenges (i.e., DRS15-challenge), and self-perceived cognitive failures (i.e., CFQ) measures

	t	df	*p*	Females	Males	d	95% confidence interval of the difference	
							Lower	Upper
Flourishing	− 2.203	178	0.029	M = 46.74 (DS = 5.4)	M = 48.5 (DS = 5.15)	− 0.3319	− 0.630	− 0.0323
Family-satisf	− 2.739	164	0.007	M = 8.06 (DS = 2.2)	M = 8.9 (DS = 1.4)	− 0.4284	− 0.740	− 0.1146
DRS15-tot	− 1.823	178	0.070	M = 27.8 (DS = 4.9)	M = 29.09 (DS = 4.5)	− 0.2746	− 0.572	0.0237
DRS15-commitment	− 1.324	178	0.187	M = 10.55 (DS = 2)	M = 10.94 (DS = 1.8)	− 0.1994	− 0.495	0.0976
DRS15-control	− 3.018	178	0.003	M = 10.4 (DS = 2.5)	M = 11.44 (DS = 2.02)	− 0.4546	− 0.755	− 0.1518
DRS15-challenge	0.286	178	0.775	M = 6.84 (DS = 2.6)	M = 6.73 (DS = 2.8)	0.0431	− 0.252	0.3383
CFQ	0.836	178	0.404	M = 29.43 (DS = 11.1)	M = 27.95 (DS = 12.6)	0.1260	− 0.170	0.4214

## Discussion

The current study primarily intended to explore the role played by a set of psychological characteristics (i.e., satisfaction with family connections, psychological hardiness, self-reported cognitive function, and crystallized intelligence) in predicting flourishing in the late adult lifespan. Moreover, to our knowledge, this is the first study investigating the impact of the DRS-15 as a measure of resilience in the last decades of life. Overall, the emerging findings let us provide insights into the interplay between satisfaction with the relations established with family members, metacognitive efficiency, resilience, and eudaimonic well-being in the last decades of life. Specifically, as expected [22, 29], higher flourishing was associated with more developed resilience, that is, older individuals being more optimistic for their future, engaged in their social network, and having a gratifying and purposeful life also reported to be more prone to face actively the adversities and stressors in their daily life, managing and controlling them at the best of their possibilities. Moreover, extending previous studies (e.g., [14, 17, 25]), a significant relationship has been also found between the flourishing index and the self-reported degree of satisfaction with family ties, suggesting that the dynamics established by the intrafamilial relations are crucial to feel well and have a purposeful life. Besides, as already reported by Fastame and Melis [26], both perceived and actual cognitive functioning evaluations are associated with flourishing in late adulthood, that is, older people being self-confident in the good functioning of their mind believed to easily manage their daily activities and they felt capable to conduct a life still valuable and purposeful. In addition, as expected [15], age was not associated with flourishing, suggesting that in the last 4 decades of life, that dimension of psychological well-being does not seem to be influenced by chronological age.

However, the most innovative finding emerging from this investigation is that satisfaction with family relations, psychological hardiness, and self-reported metacognitive efficiency predicted 30% of the variance in the flourishing condition. Keeping in mind the exploratory nature of this study, the satisfaction with family ties [17] and the metacognitive measure [26] could be expected to be significant predictors of flourishing, whereas the evidence on the role played by psychological hardiness is innovative since not previous research explored it. Overall, the results of this study suggest that feeling capable of managing adversities and daily problems thanks to the good functioning of one’s mind, being supported and gratified by one’s family, and exhibiting the attitude to cope proactively with possible stressors is crucial to enhance eudaimonic well-being in late adult lifespan. It must be noticed that consistent with previous studies [12, 15], flourishing was more developed in males than in females, and following Hone et al. [8], our male participants reported a very high level of flourishing. Moreover, extending previous evidence [22], psychological hardiness was more preserved in male participants than in females, whereas, as expected [30], gender did not impact self-perceived cognitive function. Altogether, extending previous evidence (e.g., [5, 13, 14, 20]), the current findings let us speculate that a very high level of flourishing, combined with a good degree of confidence in one’s mental functioning and a supportive social capital could represent a crucial protective factor for successful aging and perhaps to contrast the mortality risk.

From an applied viewpoint, the current findings encourage the implementation of specific interventions aimed both at enhancing the metacognitive efficiency and social participation of older people (e.g., this would also have beneficial effects on the cognitive reserve), and at strengthening (and if necessary at reorganizing) the family relationships. Altogether, these combined psychosocial programs should foster different dimensions of eudaimonic and hedonic well-being, and quality of life in the last decades of life.

It is worth noting, however, that caution is needed in generalizing these outcomes since the study has several limitations relative, for instance, to the sample size, the battery of tests used, the cross-sectional nature of the investigation, and the fact that our participants were only community dwellers. Therefore, future research should overcome these issues, replicating the study with wider samples of participants both community dwellers and institutionalized. Moreover, to appreciate the developmental trends associated with the development of flourishing, resilience, and cognitive functioning in the last decades of life, the conduction of longitudinal studies based on the administration of a wider battery of tests is desirable.

## Data Availability

The data that support the findings of this study are not publicly available due to privacy or ethical restrictions.
